# Mortality in patients with secondary peritonitis treated by primary closure or vacuum-assisted closure: nationwide register-based cohort study

**DOI:** 10.1093/bjsopen/zraf118

**Published:** 2025-11-12

**Authors:** Pooya Rajabaleyan, Lasse Kaalby, Ulrik Deding, Issam al-Najami, Mark Bremholm Ellebæk

**Affiliations:** Research Unit for Surgery, Odense University Hospital, Odense, Denmark; Faculty of Health Sciences, University of Southern Denmark, Odense Denmark; Research Unit for Surgery, Odense University Hospital, Odense, Denmark; Faculty of Health Sciences, University of Southern Denmark, Odense Denmark; Research Unit for Surgery, Odense University Hospital, Odense, Denmark; Faculty of Health Sciences, University of Southern Denmark, Odense Denmark; Research Unit for Surgery, Odense University Hospital, Odense, Denmark; Faculty of Health Sciences, University of Southern Denmark, Odense Denmark; Research Unit for Surgery, Odense University Hospital, Odense, Denmark; Faculty of Health Sciences, University of Southern Denmark, Odense Denmark

**Keywords:** open abdomen, primary abdominal closure

## Abstract

**Background:**

Secondary peritonitis caused by gastrointestinal perforation is associated with significant morbidity and mortality. Effective management includes surgical source control, antibiotic therapy, and intensive resuscitation. The choice between primary abdominal closure (PAC) and vacuum-assisted closure (VAC) in the management of secondary peritonitis remains a subject of debate.

**Methods:**

This Danish nationwide register-based cohort study included patients undergoing emergency surgery for secondary peritonitis from perforation of the small intestine, colon, or rectum between 2007 and 2021 who were treated with either PAC or VAC. Data were extracted from national registries, including the Danish Register of Cause of Death and the Danish National Patient Registry. The primary outcome was overall all-cause mortality; secondary outcomes were all-cause mortality at 30 days, 90 days, and 1 year.

**Results:**

In all, 13 898 patients were included (1017 in the VAC group, 12 881 in the PAC group). VAC-treated patients had significantly higher Charlson Co-morbidity Index scores and were slightly younger. In the subgroup with available laboratory data, VAC-treated patients also presented with more severe biochemical derangements, including elevated C-reactive protein, leukocytes, bilirubin, and lactate, as well as lower haemoglobin, suggesting a higher baseline severity of illness. The overall risk-stratified mortality rate (RSMR) was 49.1% for VAC and 52.0% for PAC (*P* = 0.222). The 30-day mortality rate was 16.9% in both the VAC and PAC groups, with RSMR of 17.4% and 18.3%, respectively (*P* = 0.656). At 90 days, mortality was 24.3% and 22.5% in the VAC and PAC groups, respectively, with a corresponding RSMR of 23.2% and 24.2% (*P* = 0.437). One year after surgery, the mortality rate was 31.3% for VAC and 29.5% for PAC, with a corresponding RSMR of 30.3% and 31.6% (*P* = 0.346).

**Conclusion:**

This nationwide cohort study revealed no significant differences in mortality between PAC and VAC in patients with secondary peritonitis at any of the designated time points. Demographic and laboratory data suggest that VAC-treated patients had a higher baseline severity of illness.

## Teaser

This nationwide Danish register-based cohort study compared mortality outcomes in 13 898 patients undergoing surgery for secondary peritonitis between primary abdominal closure (PAC) and vacuum-assisted closure (VAC). No significant differences were found in overall or time-specific (30-day, 90-day, and 1-year) mortality between the two groups. These findings suggest that PAC and VAC are comparable in terms of mortality outcomes in the management of secondary peritonitis.

## Introduction

Secondary peritonitis often originates from gastrointestinal tract perforation^1^. It is a significant global health concern, contributing to approximately 896 000 deaths annually^2^. Annually, this life-threatening condition accounts for 20 million years of life lost and 25 million disability-adjusted life years worldwide^2^. Without timely and adequate source control, secondary peritonitis can rapidly escalate to sepsis^3^. Intra-abdominal sepsis is the second leading cause of sepsis-related mortality in critically ill patients^4^.

The cornerstone of treatment of secondary peritonitis includes early source control, broad-spectrum antibiotic therapy, and intensive supportive care^3,5–9^. Source control aims to eliminate infection, prevent further contamination, and restore physiological stability through surgical intervention, antibiotics, drainage, and haemodynamic support.^3,10^ Failure of source control, whether due to inadequate drainage, anastomotic leakage, persistent intra-abdominal contamination, or compartment syndrome, can result in ongoing infection, sepsis, multiorgan failure, and increased mortality^3,11,12^.

Surgical intervention plays a pivotal role, with two commonly used approaches to abdominal closure: primary abdominal closure (PAC) and vacuum-assisted closure (VAC)^5–9^. The decision between these techniques is influenced by factors such as haemodynamic stability, intra-abdominal pressure, ongoing infection, and the feasibility of definitive closure^5–7,9,13^. PAC avoids scheduled reoperations but carries the risk of delayed treatment for ongoing abdominal sepsis or abdominal compartment syndrome, which can be difficult to detect in critically ill patients^14–17^. Delays in addressing these complications may increase morbidity and mortality^3,7^. In contrast, VAC leaves the peritoneal cavity open, allowing for planned reassessments to detect complications early and intra-abdominal pressure management^3,5–9,18^. VAC may also reduce peritoneal cytokines and inflammatory mediators, potentially mitigating severe sepsis and septic complications. Although animal models and *in silico* studies support the reduction of peritoneal cytokines and inflammatory mediators, human studies have yet to confirm these findings^19–21^ . Despite these advantages, VAC has been associated with longer hospitalization, increased catabolic drainage, delayed closure, enteroatmospheric fistula formation, and ventral hernias^3,5–9^. However, some evidence suggests that enteroatmospheric fistula development is linked more to underlying peritoneal pathology than VAC itself^22,23^.

Mortality rates associated with these approaches vary considerably. Among patients treated with VAC, 30-day mortality rates can reach 16.91%^24–26^ and overall mortality can rise as high as 85.11%^26–44^. In contrast, PAC-treated patients have 30-day mortality rates up to 22.67%^24,30,45–47^ and overall mortality reportedly ranges up to 58.82%^30,32,48–53^. Key independent risk factors influencing mortality in these patients include late-onset hospital-acquired infections, diffuse peritonitis, sepsis, septic shock, advanced age, malnutrition, liver failure, congestive heart failure, antimicrobial resistance, and failure to control the infection source^54–57^.

Given the high mortality rates associated with secondary peritonitis, and to aid clinical decision-making towards the most effective closure technique, the aim of this study is to provide insights into both short- and long-term mortality outcomes of VAC *versus* PAC. Using national registry-based data, the 30-day, 90-day, 1-year, and overall all-cause mortality rates associated with the two treatment modalities were assessed, and predictors of mortality were identified.

## Methods

### Study design

This was a nationwide register-based cohort study of Danish patients who underwent emergency surgery with source control followed by either VAC or PAC for secondary peritonitis arising from the small intestine, colon, or rectum. The study encompassed a broad range of underlying conditions leading to secondary peritonitis, such as Meckel’s diverticulum, small bowel volvulus, anastomotic leakage, bowel perforations (in the small intestine, colon, or rectum), diverticulitis with perforation, colonic volvulus, small or large bowel obstruction, hernia with bowel obstruction, ischaemic colitis, and perforation following colonoscopy (*Table S1*). Patients were included from 2007 to 2021, and those with missing data for any covariates were excluded from the complete case analysis.

This study was registered with the Danish Data Protection Agency (22/5636), and data access and handling were approved by and stored at Statistics Denmark. In accordance with Danish law, registry-based studies do not require consent from the patients included in the study.

### Outcomes

The primary outcome was overall all-cause mortality; secondary outcomes were all-cause mortality at 30 days, 90 days, and 1 year. The study also identified independent predictors of mortality.

### Data sources and covariates

In Denmark, each resident is assigned a unique personal identification number, the Central Personal Registration number, enabling complete linkage across various registries. Mortality data were obtained from the Danish Register of Cause of Death^58^. Using the International Classification of Diseases 10th revision codes, the Danish National Patient Registry^59^, which covers all interactions with hospital-based healthcare services, was searched to identify patients with conditions leading to secondary peritonitis of the lower gastrointestinal tract and linked with surgical procedure codes (for detailed combinations, see *Table S1*). Information on patient educational levels and incomes was sourced from the Population’s Educational Register^60^ and the Income Statistics Register^61^.

Demographic and baseline covariates were gathered from the Danish National Patient Registry^59^, the Population’s Education Register^60^, and the Income Statistics Register^61^. These covariates included sex, age groups (categorized as < 45, 45–54, 55–64, 65–74, and > 74 years), Charlson Co-morbidity Index (CCI; 0, 1, 2, or 3), educational attainment was categorised as elementary school (compulsory schooling up to 9th grade), high school (general upper secondary education, typically three years, academically oriented), vocational school (profession-oriented upper secondary programs of 2–4 years combining classroom training and apprenticeship), short education (short-cycle tertiary education, typically ≤2 years, such as academy profession programmes and vocational diplomas), and higher education (medium- and long-cycle tertiary education, including bachelor’s, master’s, and PhD degrees). Income was classified into inflation-adjusted 5-year average income quintiles, ranging from quintile 1 (lowest 20% of the population) to quintile 5 (highest 20%)^62^.

A range of blood sample test results were included as covariates and indicators of preoperative disease severity. These included C-reactive protein (CRP), leukocytes, lymphocytes, neutrophils, haemoglobin, albumin, estimated glomerular filtration rate (eGFR), bilirubin, platelets, lactate, and the neutrophil-to-lymphocyte ratio (NLR). Blood sample data were retrieved from national laboratory databases and were available for patients treated after 2016. Only preoperative blood sample test results were analysed, representing the average of all available measurements within 48 hours before surgery.

### Statistical analysis

Descriptive statistics of baseline characteristics are presented as frequencies and percentages and were analysed using Pearson’s χ^2^ test.

The primary analysis compared mortality between the VAC and PAC groups using risk-stratified mortality rates (RSMRs) at 30 days, 90 days, 1 year, and overall. The RSMR was based on a multivariate logistic regression model, accounting for the influence of covariates on mortality rates. The RSMR is reported per 100 observations, including the 95% confidence interval (c.i.). RSMR proportions were compared using a *Z* test. Kaplan–Meier curves were used to illustrate survival data over time.

As a secondary analysis, overall mortality was assessed using a multivariate Cox proportional hazards regression model, adjusting for age, sex, procedure type, income, education, and CCI. Both univariate and multivariate models were performed for each outcome, and hazard ratios (HRs) with 95% confidence intervals are reported.

Blood sample test results were analysed descriptively to compare distributions between the VAC and PAC groups, with median values reported due to skewed distributions. In sensitivity analysis, a multivariate Cox model was used in the subgroup of patients with available laboratory data (*n* = 2827) to assess the prognostic value of preoperative inflammatory markers, specifically C-reactive protein (CRP) and the neutrophil-to-lymphocyte ratio (NLR). These markers were prioritized due to their established roles in systemic inflammation and the low proportion of missing data.

*P* < 0.05 was considered statistically significant. All data management and statistical analyses were conducted using Stata Software Release 18 (StataCorp, College Station, TX, USA).

## Results

### Baseline characteristics

The study included 13 898 patients, divided into two groups: those who underwent VAC (1017) and those who underwent PAC (12 881). No patients were excluded from the primary analysis due to missing data on key variables. Patient characteristics are presented in (*Table 1*).

**Table 1:** Demographic data: comparison between VAC and PAC

	VAC (*n* = 1017)	PAC (*n* = 12 881)	Total (*n* = 13 898)	P*
**Age group**				
< 45 years	75 (7.4%)	1057 (8.2%)	1132 (8.2%)	< 0.001
45–54 years	115 (11.3%)	1294 (10.1%)	1409 (10.1%)	
55–64 years	196 (19.3%)	2293 (17.8%)	2489 (17.9%)	
65–74 years	353 (34.7%)	3507 (27.2%)	3860 (27.8%)	
> 74 years	278 (27.3%)	4730 (36.7%)	5008 (36.0%)	
**Sex**				
Male	542 (53.3%)	5926 (46.0%)	6468 (46.5%)	< 0.001
Female	475 (46.7%)	6955 (54.0%)	7430 (53.5%)	
**Highest completed education**				
Elementary	418 (41.1%)	5098 (39.6%)	5516 (39.7%)	0.078
Vocational/high school	399 (39.2%)	4794 (37.2%)	5193 (37.4%)	
Short–intermediate	143 (14.1%)	1831 (14.2%)	1974 (14.2%)	
Higher	32 (4.9%)	621 (4.8%)	653 (4.7%)	
Missing information	25 (2.5%)	537 (4.2%)	562 (4.0%)	
**Income quintiles (%)**				
1	220 (21.6%)	3195 (24.8%)	3415 (24.6%)	0.156
2	260 (25.6%)	3221 (25.0%)	3481 (25.1%)	
3	271 (26.7%)	3210 (24.9%)	3481 (25.1%)	
4	260 (25.6%)	3220 (25.0%)	3480 (25.0%)	
5	6 (0.6%)	35 (0.3%)	41 (0.3%)	
**CCI score (%)**				
0	365 (35.9%)	5313 (41.3%)	5678 (40.9%)	0.011
1	117 (11.5%)	1371 (10.6%)	1488 (10.7%)	
2	301 (29.6%)	3467 (26.9%)	3768 (27.1%)	
3	234 (23.0%)	2730 (21.2%)	2964 (21.3%)	
**Year of surgery (%)**				
2007–2014	396 (5.2%)	7214 (94.8%)	7610 (55.0%)	< 0.001
2015–2021	621 (9.9%)	5667 (90.1%)	6296 (45.2%)	
**Status (%)**				
Alive	518 (50.9%)	6178 (48.0%)	6696 (48.2%)	0.068
Dead	499 (49.1%)	6703 (52.0%)	7202 (51.8%)	
**Time in study (days)**				
Median	798	982	969	
Mean	1225	1532	1509	

Values are *n* (%) unless otherwise stated. Educational attainment: elementary, high school (general upper secondary), vocational school (profession-oriented upper secondary, 2–4 years), short education (short-cycle tertiary, ≤2 years), higher education. Income: quintiles 1–5, from lowest (20%) to highest (20%). VAC, vacuum-assisted closure; PAC, primary abdominal closure; CCI, Charlson Co-morbidity Index. *Pearson’s χ^2^ test.

The largest age group was that aged > 74 years (36.0%), followed by the group aged 65–74 years (27.8%). Patients aged > 65 years comprised 62.1% of the VAC group and 64.0% of the PAC group, indicating a slightly younger VAC population (*P* < 0.001). Men accounted for 46.5% of the total cohort, 53.3% of the VAC group, and 46.0% of the PAC group (*P* < 0.001).

There was no significant difference (*P* = 0.011) in the distribution of CCI scores between the VAC and PAC groups: CCI 0, 35.9% *versus* 41.3%, respectively; CCI 1, 11.5% *versus* 10.6%, respectively; CCI 2, 29.6% *versus* 26.9%, respectively; and CCI 3, 23.0% *versus* 21.2%, respectively.

The proportion of patients treated with VAC increased over time, from 5.2% in 2007–2014 to 9.9% in 2015–2021, whereas the use of PAC decreased from 94.8% in 2007–2014 to 90.1% in 2015–2021 (*P* < 0.001).

### Mortality outcomes

**Fig. 1:**
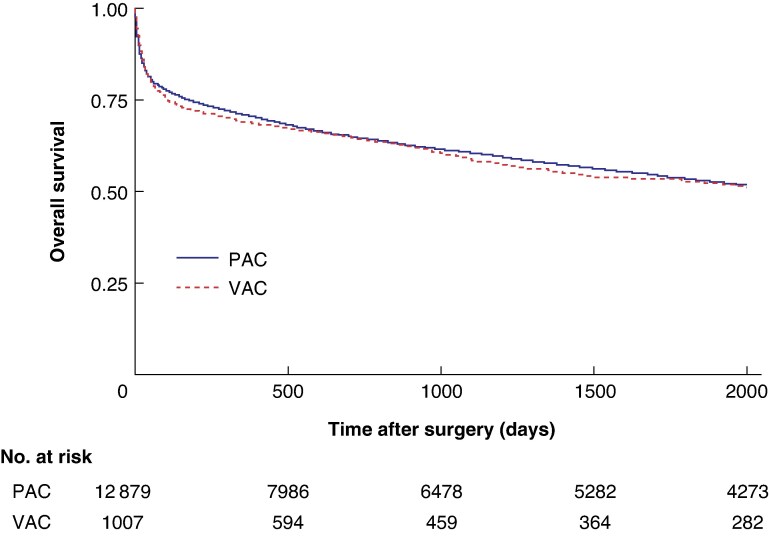
Kaplan–Meier survival estimates for the PAC and VAC groups Overall survival over time is shown. PAC, primary abdominal closure; VAC, vacuum-assisted closure.

Mortality rates are summarized in *Table 2*, with Kaplan–Meier curves shown in *Fig. 1*. Crude mortality rates and RSMRs (adjusted for covariates) are reported below.

**Table 2:** Risk-stratified mortality: comparison between VAC and PAC

Mortality	Observed (*n*)	Expected (*n*)	Total (*n*)	Crude MR (per 100 observations)	RSMR* (per 100 observations)	*P*†
**30 days**						
PAC	2183	2024	12 881	16.95	18.28 (17.52, 19.06)	0.656
VAC	172	168	1017	16.91	17.35 (14.90, 20.14)	
**90 days**						
PAC	2893	2695	12 881	22.46	24.23 (23.34, 25.16)	0.437
VAC	248	242	1017	24.34	23.16 (20.37, 26.23)	
**1 year**						
PAC	3795	3555	12 881	29.46	31.59 (30.59, 31.61)	0.346
VAC	318	311	1017	31.27	30.26 (27.03, 33.78)	
**Overall**						
PAC	6703	6298	12 881	52.04	55.15 (53.84, 56.49)	0.222
VAC	499	484	1017	49.07	53.42 (48.84, 58.33)	

^*^Values in parentheses are 95% confidence intervals. RSMRs were derived from multivariate logistic regression models adjusting for covariates. The significance of differences between RSMRs was tested using *Z*-test. VAC, vacuum-assisted closure; PAC, primary abdominal closure; MR, mortality rate; RSMR, risk-stratified mortality rate. †Z-test.

At 30 days, the crude mortality rate was 172 of 1017 patients (16.9%) in the VAC group and 2183 of 12 881 patients (16.9%) in the PAC group. The RSMR was 17.4% (95% c.i. 14.9 to 20.1) for VAC and 18.3% (95% c.i. 17.5 to 19.1) for PAC (*P* = 0.656).

At 90 days, 248 VAC patients (24.3%) and 2893 PAC patients (22.5%) had died. The RSMRs were 23.2% (95% c.i. 20.4 to 26.2) for VAC and 24.2% (95% c.i. 23.3 to 25.2) for PAC (*P* = 0.437).

At 1 year, the crude mortality rate was 318 of 1017 (31.3%) in the VAC group and 3795 of 12 881 (29.5%) in the PAC group. The corresponding RSMRs were 30.3% (95% c.i. 27.0 to 33.8) and 31.6% (95% c.i. 30.6 to 31.6), respectively (*P* = 0.346).

Overall, 499 patients (49.1%) in the VAC group and 6703 (52.0%) in the PAC group died during the study period. The RSMRs were 53.4% (95% c.i. 48.8 to 58.3) for VAC and 55.2% (95% c.i. 53.8 to 56.5) for PAC (*P* = 0.222).

The median follow-up time was 798 days for the VAC group and 982 days for PAC; mean follow-up durations were 1225 and 1532 days, respectively.

### Predictors of mortality

Cox regression analysis (*Table 3*) identified age, co-morbidity, sex, income, and education as significant predictors of mortality. Patients aged > 74 years had the highest risk of mortality (HR 8.11; 95% c.i. 6.74 to 9.75; *P* < 0.0001). Compared with a CCI score of 0, CCI scores of 1 (HR 1.88), 2 (HR 1.89), and 3 (HR 3.14) were all associated with increased mortality (*P* < 0.001 for all).

**Table 3:** Cox proportional hazards for overall mortality: comparison between VAC and PAC

	Hazard ratio	Standard error	P*
**Procedure**			
Univariate			
PAC	Reference		
VAC	1.04 (0.95, 1.14)	0.049	0.392
Multivariate			
PAC	Reference		
VAC	1.05 (0.96, 1.15)	0.05	0.362
**Age group**			
< 45 years	Reference		
45–54 years	2.24 (1.81, 2.76)	0.24	< 0.001
55–64 years	3.46 (2.86, 4.18)	0.34	< 0.001
65–74 years	4.39 (3.65, 5.29)	0.42	< 0.001
> 74 years	8.11 (6.74, 9.75)	0.76	< 0.001
**Sex**			
Male	Reference		
Female	0.8 (0.76, 0.84)	0.05	< 0.001
**Income quintiles**			
1	Reference		
2	0.91 (0.86, 0.97)	0.03	0.004
3	0.77 (0.72, 0.83)	0.03	< 0.001
4	0.58 (0.54, 0.64)	0.03	< 0.001
**Highest completed education**			
Elementary	Reference		
Vocational/high school	0.90 (0.86, 0.96)	0.03	0.001
Short–intermediate	0.87 (0.8, 0.94)	0.04	0.001
Higher	0.92 (0.8, 1.06)	0.06	0.242
**CCI score**			
0	Reference		
1	1.88 (1.73, 2.05)	0.08	< 0.001
2	1.89 (1.77, 2.02)	0.06	< 0.001
3	3.14 (2.94, 3.35)	0.11	< 0.001

Values in parentheses are 95% confidence intervals. Univariate and multivariate Cox regression results for overall mortality are presented. The multivariate model was adjusted for procedure type, age, sex, income, education, and CCI. Educational attainment: elementary, high school (general upper secondary), vocational school (profession-oriented upper secondary, 2–4 years), short education (short-cycle tertiary, ≤2 years), higher education. Income: quintiles 1–5, from lowest (20%) to highest (20%). VAC, vacuum-assisted closure; PAC, primary abdominal closure; CCI, Charlson Co-morbidity Index. *Pearson’s χ2 test.

Women had a lower mortality risk than men (HR 0.80; 95% c.i. 0.76 to 0.84; *P* < 0.001). Higher income levels and intermediate education (vocational, short–intermediate) were associated with a reduced risk of mortality, whereas higher education had no significant effect.

Supplementary analyses (*Table S2*) showed no significant association between the VAC procedure and mortality (odds ratio 1.10; 95% c.i. 0.90 to 1.36; *P* = 0.25). Age, sex, income, education, co-morbidity, and year of surgery remained significant predictors of mortality.

### Preoperative laboratory parameters and sensitivity analysis

Preoperative blood sample results differed between groups (*Table 4*; *Figs S3–4*). Mean CRP levels were higher in the VAC than PAC group (151.62 *versus* 100.42 mg/l, *P* < 0.001). Similarly, VAC patients had higher mean leukocyte counts (12.82 *versus* 12.20 × 10^9^/L; *P* = 0.036), bilirubin (15.67 *versus* 13.20 µmol/l; *P* = 0.016), lactate (2.34 *versus* 1.89 mmol/l; *P* < 0.001), and eGFR (76.50 *versus* 69.74 ml/min/1.73 m^2^; *P* < 0.001) than PAC patients. Both mean haemoglobin (7.47 *versus* 7.75 g/dl; *P* < 0.001), and mean albumin (28.99 *versus* 32.43 g/l; *P* < 0.001) were significantly lower in the VAC than PAC group. NLR values were similar in the VAC and PAC groups (11.98 *versus* 12.05, respectively; *P* = 0.768).

**Table 4:** Blood sample test results analysis: comparison between VAC and PAC

	VAC	PAC	Total	P*
**CRP**				
Total no. of samples	629	6863	7492	< 0.001
Median concentration (mg/L)	129.95	63.30		
Mean concentration (mg/L)	151.62	100.42		
**Leukocytes**				
Total no. of samples	545	6715	7260	0.036
Median count (× 10^9^/l)	11.65	11.00		
Mean count (× 10^9^/l)	12.82	12.20		
**Lymphocytes**				
Total no. of samples	280	3830	4110	0.278
Median count (× 10^9^/l)	0.975	1.03		
Mean count (× 10^9^/l)	1.14	1.20		
**Neutrophils**				
Total no. of samples	229	3222	3451	0.956
Median count (× 10^9^/l)	9.37	9.11		
Mean count (× 10^9^/l)	10.45	10.30		
**Haemoglobin**				
Total no. of samples	552	6951	7503	< 0.001
Median concentration (mmol/l)	7.40	7.80		
Mean concentration (mmol/l)	7.47	7.75		
**Albumin**				
Total no. of samples	625	6767	7392	< 0.001
Median concentration (g/l)	29.00	33.00		
Mean concentration (g/l)	28.99	32.43		
**eGFR**				
Total no. of samples	621	6652	7273	< 0.001
Median eGFR (ml/min/1.73 m^2^)	68.00	64.48		
Mean eGFR (ml/min/1.73 m^2^)	76.50	69.74		
**Bilirubin**				
Total no. of samples	504	5882	6386	0.016
Median concentration (µmol/l)	11.00	10.50		
Mean concentration (µmol/l)	15.67	13.20		
**Platelets**				
Total no. of samples	584	6350	6934	0.496
Median count (× 10^9^/l)	285.50	308.71		
Mean count (× 10^9^/l)	286.00	311.02		
**Lactate**				
Total no. of samples	396	3739	4135	< 0.001
Median concentration (mmol/l)	1.70	1.30		
Mean concentration (mmol/l)	2.34	1.89		
**NLR**				
Total no. of samples	185	2815	3000	0.768
Median	8.01	8.70		
Mean	11.98	12.05		

VAC, vacuum-assisted closure; PAC, primary abdominal closure; CRP, C-reactive protein; eGFR, estimated glomerular filtration rate; NLR, neutrophil-to-lymphocyte ratio. *Mann–Whitney *U* test for skewed continuous variables.

A sensitivity analysis was conducted in patients with available CRP and NLR data (2827 patients). In multivariate analysis (*Table 5*), both CRP (HR 1.00; 95% c.i. 1.00 to 1.00; *P* < 0.001) and NLR (HR 1.01; 95% c.i. 1.01–1.02; *P* < 0.001) were independently associated with overall mortality. However, the effect size per unit increase in CRP was minimal.

**Table 5:** Sensitivity analysis using CRP and NLR: Cox proportional hazards analysis for overall mortality comparing VAC and PAC

	Hazard ratio*	Standard error	*P*
**Procedure**			
PAC	Reference		
VAC	1.10 (0.88, 1.39)	0.13	0.400
**Age group**			
< 45 years	Reference		
45–54 years	3.11 (1.83, 5.28)	0.84	< 0.001
55–64 years	3.73 (2.28, 6.10)	0.94	< 0.001
65–74 years	5.32 (3.30, 8.58)	1.30	< 0.001
> 74 years	8.66 (5.39, 13.92)	2.10	< 0.001
**Sex**			
Male	Reference		
Female	0.71 (0.63, 0.79)	0.04	< 0.001
**Income quintiles**			
1	Reference		
2	0.96 (0.83, 1.12)	0.07	0.614
3	0.83 (0.71, 0.97)	0.07	0.043
4	0.67 (0.56, 0.81)	0.06	< 0.001
**Highest completed education**			
Elementary	Reference		
Vocational/high school	0.97 (0.86, 1.09)	0.06	0.619
Short–intermediate	0.78 (0.65, 0.94)	0.07	0.010
Higher	0.81 (0.60, 1.09)	0.12	0.190
**CCI score**			
0	Reference		
1	1.93 (1.59, 2.35)	0.19	< 0.001
2	1.87 (1.60, 2.17)	0.15	< 0.001
3	3.03 (2.61, 3.53)	0.24	< 0.001
**Blood samples**			
NLR	1.01 (1.01, 1.02)	0.00	< 0.001
Average CRP concentration	1.00 (1.00, 1.00)	0.00	< 0.001

^*^Values in parentheses are 95% confidence intervals. The number of subjects included in the analysis was 2827; the number of deaths was 1289. This table presents multivariate Cox regression results for overall mortality. The multivariate model was adjusted for procedure type, age, sex, income, education, CCI score, NLR, and CRP. Educational attainment: elementary, high school (general upper secondary), vocational school (profession-oriented upper secondary, 2–4 years), short education (short-cycle tertiary, ≤2 years), higher education. Income: quintiles 1–5, from lowest (20%) to highest (20%). CRP, C-reactive protein; NLR, neutrophil-to-lymphocyte ratio; VAC, vacuum-assisted closure; PAC, primary abdominal closure; CCI, Charlson Co-morbidity Index.

## Discussion

This nationwide register-based cohort study represents the largest investigation to date on mortality and predictors of mortality in patients with secondary peritonitis from the small intestine, colon, or rectum treated with either PAC or VAC. Despite the suggested advantages of VAC in managing severe peritonitis, no significant differences in mortality were found between the two groups at 30 days, 90 days, 1 year, or overall. VAC-treated patients had significantly higher CCI scores and a slightly younger age distribution. In the subset of patients with available blood sample results, VAC-treated patients had significantly elevated CRP, leukocyte counts, bilirubin, and lactate levels, along with lower haemoglobin and albumin levels compared with the PAC group. Key predictors of increased mortality included advanced age, male sex, higher CCI scores, lower income, and lower vocational/intermediate education, as well as elevated NLR and mean CRP concentration.

The management of patients with severe secondary peritonitis is inherently complex^3,5–9,13,18,63,64^. A fundamental question remains as to whether the abdomen should be managed as an ‘open abscess’ using VAC systems or closed with PAC and reoperated on demand^65,66^. Addressing this issue is crucial to optimizing surgical strategies, because the potential benefits of the chosen method may be substantial.

To contextualize the PAC findings, non-comparative studies involving exclusively PAC-treated patients were reviewed. In a randomized controlled trial, van Ruler *et al*.^53^ included patients with secondary peritonitis of gastrointestinal origin, all of whom received PAC and were randomized to either planned or on-demand relaparotomy. The study found no significant difference in 1-year mortality between planned relaparotomy (36%) and on-demand relaparotomy (29%)^53^. A retrospective study by Bensignor *et al*.^48^, which included 191 patients treated with PAC only for postoperative peritonitis, showed a 14% overall mortality rate. The reported 1-year mortality rates are in line with those observed in the PAC group (29.5%). However, the overall mortality in the PAC group (52.0%) was substantially higher, possibly reflecting the longer follow-up time.

Non-comparative studies evaluating VAC have reported relatively consistent results. In a 2015 systematic review and meta-analysis on non-trauma patients treated with an open abdomen, Atema *et al*.^67^ reported VAC-associated in-hospital mortality rates ranging from 21.5 to 30.0% depending on the specific VAC approach used. In their retrospective analysis of 438 patients treated for various abdominal emergencies with VAC, Gasser *et al*.^25^ reported mortality rates of 14% at 30 days, increasing to 31% at 1 year. In comparison, in the present study, the 30-day, 90-day, and 1-year mortality rates in the VAC group were 16.9%, 24.3%, and 31.3%, respectively, aligning with these previous reports.

Three of the largest comparative studies to date have reported divergent mortality outcomes between VAC and PAC in secondary peritonitis. In their retrospective analysis, Bleszynski *et al*.^24^ reported a significantly lower in-hospital mortality for VAC of 22.8%, compared with 38.6% for PAC, in patients undergoing urgent laparotomy for severe sepsis or septic shock. This suggests a possible survival advantage with VAC. Although no significant difference was observed in the cohort, VAC was more often used in patients with higher co-morbidity and elevated inflammatory markers.

Further, a retrospective comparative study including 176 VAC and 139 PAC patients with secondary peritonitis of lower gastrointestinal origin found no significant difference in 30-, 90-, or 1-year mortality^68^. Although VAC was associated with fewer surgical complications and reduced need for reoperations, it was also linked to longer intensive care unit stays. These mortality findings are consistent with those observed in the present study.

In contrast, a third comparative study identified higher in-hospital mortality among VAC-treated patients. Kao *et al.*^32^ reported their retrospective propensity-matched study of non-trauma patients with secondary peritonitis, comparing 203 patients treated with VAC to 331 patients treated with PAC. After propensity score matching, in-hospital mortality was significantly higher for VAC-treated patients than PAC-treated patients (22.5% *versus* 11.7%, respectively). Importantly, the VAC-treated patients in that study were significantly more severely ill, older, and more often transferred from other facilities due to the complexity of their conditions^32^. A greater proportion of these patients required inotropic support and blood transfusions, factors that indicated elevated mortality rates. Together, these findings suggest that although VAC may offer therapeutic benefits in managing secondary peritonitis, its advantages can be diminished by the severity of the underlying condition, highlighting the need for careful patient selection in future research.

In the present cohort, significantly elevated CRP, leukocyte counts, bilirubin, and lactate levels, as well as lower haemoglobin and albumin levels, were observed in VAC-treated patients compared with PAC-treated patients. In addition, VAC patients had higher CCI scores, with a greater proportion presenting with severe co-morbidities (CCI score ≥2). These findings indicate a heightened inflammatory and systemic response, likely reflecting the selection of more critically ill patients for VAC. Despite these differences, mortality remained comparable across all time points, suggesting that VAC may be a viable option for patients with secondary peritonitis, even in the presence of increased illness severity.

Regarding socioeconomic factors, variables such as income and education were included in the multivariate analysis to account for potential confounding related to access to care, patient management, and health outcomes. Lower income and education levels are often associated with reduced access to high-quality care, delayed treatment, and poorer long-term outcomes^69^. Significant associations between these covariates and mortality were identified, suggesting that disparities in socioeconomic status may have contributed to the variation in outcomes observed in the cohort. These findings align with previous research highlighting socioeconomic status as an independent predictor of postoperative complications and mortality^70^.

The present study was affected by several limitations inherent in its register-based design. Unmeasured or residual confounding factors, particularly those related to a patient’s physiological state at the index operation and postoperative complications, such as abscesses and enteroatmospheric fistulas, could not be fully retrieved due to incomplete registration. These factors, if available, could have provided valuable insights into the characteristics of the population studied and outcomes beyond mortality.

Furthermore, blood sample data were only available for patients treated after 2016, resulting in substantial missingness for earlier cases. As a result, laboratory parameters such as CRP and NLR could not be included in the primary adjusted models and were instead confined to sensitivity and subgroup analyses. Although these biomarkers offer some indication of systemic inflammation, they do not constitute a comprehensive assessment of preoperative physiological status.

Despite these limitations, the use of a structured national register provided a consistent and broad data source, although the retrospective nature of the data limits its completeness and clinical detail. In addition, the large sample size enhanced statistical power and strengthened the external validity of the findings. Notably, even with missing laboratory data, the number of patients included in the analyses remains among the highest reported in the literature to date, particularly with respect to comparative studies of VAC and PAC in secondary peritonitis.

This nationwide register-based cohort study showed no significant difference in 30-day, 90-day, 1-year, and overall all-cause mortality between patients with secondary peritonitis treated with PAC or VAC. Key predictors of increased mortality included advanced age, male sex, higher CCI, lower income, lower vocational/intermediate education, NLR, and CRP. Although the data may reflect VAC being more commonly used in critically ill patients than PAC, further research, such as randomized clinical trials, is needed to determine its potential benefits, especially in these subgroups.

## Supplementary Material

zraf118_Supplementary_Data

## Data Availability

Upon reasonable request, the data may be shared in anonymized form, provided a data processor agreement is obtained.
