# Supplementary material for: Mortality in patients with secondary peritonitis treated by primary closure or vacuum-assisted closure: nationwide register-based cohort study
Source: BJS Open. 2025 Nov 12;9(6):zraf118. doi: 10.1093/bjsopen/zraf118 (PMC12605727; doi:10.1093/bjsopen/zraf118)
Supplement: zraf118_Supplementary_Data [file zraf118_supplementary_data.docx]

# **Mortality in patients with secondary peritonitis treated by primary closure or vacuum-assisted closure: nationwide register-based cohort study**

**Authors:** Pooya Rajabaleyan, MD; Lasse Kaalby, PhD; Ulrik Deding, PhD; Issam al-Najami, MD, PhD; Mark Bremholm Ellebæk, MD, PhD

**Affiliations**

Research Unit for Surgery, Odense University Hospital, Odense, Denmark; University of Southern Denmark, Odense Denmark

**Corresponding author**

Pooya Rajabaleyan, MD

[Pooya.Rajabaleyan@skane.se](mailto:Pooya.Rajabaleyan@skane.se)

Research Unit for Surgery, Odense University Hospital, Odense, Denmark; University of Southern Denmark, Odense Denmark

**ORCID ID**; 0000-0002-6882-9427

**Supplementary Materials - Index**

| **Supplementary Figures and Tables** |  |
| --- | --- |
| Table S1 | *page 2* |
| Table S2 | *page 6* |
| Figure S3  Figure S4 | *page 7*  *page 8* |
|  |  |

**Supplementary Figures and Tables**

**Table S1: Search Strategy Combinations of ICD Codes**
This table provides an overview of the search strategy combinations of ICD codes used for identifying patients included in the study.

| **Population** | **Type** | **Yes(1)/No(2)** | **Description** | **Code** | **Accompanied By** |
| --- | --- | --- | --- | --- | --- |
| Meckel’s divertikulum | Categorical | Yes(1)/No(2) | Perforation in the wall of the intestine near the junction of the small and large intestines | LPR | DQ430 In combination with KJFB, KJFB96, KJA-, BNPA92, KJAH30, KJFH20, KJFH, KJAH33, KJFH10, KJ6B10, KJFH00 (*)Has to be registered accompanied by one of the following: DK650, DK650P, DK659, DK650M, DK650N, DK658 |
| Small bowel volvulus | Categorical | Yes(1)/No(2) | Torsion of all or parts of a segment of small bowel | LPR | DK562C In combination with KJFB-, KJFB96, KJA-, BNPA92, KJAH30, KJFH20, KJFH, KJAH33, KJFH10, KJ6B10, KJFH00 (*)Has to be registered accompanied by one of the following: DK650, DK650P, DK659, DK650M, DK650N, DK658 |
| Anastomotic leakage | Categorical | Yes(1)/No(2) | When a surgical anastomosis fails and contents leak from the anastomosis | LPR | DT813A, DT813A1, DT813A2, DT813G In combination with KJWF, KJWF00, KJFB-, KJFB20, KJFB96, KJA-, KJFB46, KJFB30, KJFB43, KJFB56, KJFB30B, KJFH96, KJFH01, BNPA92, KJAH30, KJFH20, KJFH, KJAH33, KJFH10, KJ6B10, KJFH00 |
| Small bowel, colon or rectum perforation | Categorical | Yes(1)/No(2) | Perforation of the small bowel, colon, or rectum | LPR | DK631, DK631A, DK628H In combination with KJFB-, KJFB96, KJFB20, KJFB96, KJA-, KJFB46, KJFB30, KJFB43, KJFB56, KJFB30B, KJFH96, KJFH01, KJH00, KJGB10, KJGB40, KJGB00, KJGB30, KJGB31, BNPA92, KJAH30, KJFH20, KJFH, KJAH33, KJFH10, KJ6B10, KJFH00 |
| Diverticulitis with perforation | Categorical | Yes(1)/No(2) | Inflammation in a small pouch located in the bowel leading to perforation | LPR | DK570, DK570B, DK570C, DK572B, DK572C, DK574, DK574A In combination with KJFB-, KJFB20, KJFB96, KJA-, KJFB46, KJFB30, KJFB43, KJFB56, KJFB30B, KJFH96, KJFH01, BNPA92, KJAH30, KJFH20, KJFH, KJAH33, KJFH10, KJ6B10, KJFH00 |
| Volvulus | Categorical | Yes(1)/No(2) | Condition where the bowel twists on itself, causing obstruction | LPR | DK562, DK652B In combination with KJFB-, KJFB20, KJFB96, KJA-, KJFB46, KJFB30, KJFB43, KJFB56, KJFB30B, KJFH96, KJFH01, BNPA92, KJAH30, KJFH20, KJFH, KJAH33, KJFH10, KJ6B10, KJFH00 (*)Has to be registered accompanied by one of the following: DK650, DK650P, DK659, DK650M, DK650N, DK658 |
| Ileus | Categorical | Yes(1)/No(2) | Mechanical obstruction of the bowels | LPR | DK567 In combination with KJFB, KJFB96, KJWF, KJWF00, KJFB-, KJFB20, KJFB96, KJA-, KJFB46, KJFB30, KJFB43, KJFB56, KJFB30B, KJFH96, KJFH01, BNPA92, KJAH30, KJFH20, KJFH, KJAH33, KJFH10, KJ6B10, KJFH00 (*)Has to be registered accompanied by one of the following: DK650, DK650P, DK659, DK650M, DK650N, DK658 |
| Hernia with ileus | Categorical | Yes(1)/No(2) | Abnormal exit of tissue or an organ through the abdominal wall | LPR | DK420, DK430, DK400, DK403, DK403A, DK403B, DK433, DK450, DK450C, DK450F, DK460 In combination with KJFB-, KJFB96, KJWF, KJWF00, KJFB, KJFB20, KJFB96, KJA-, KJFB46, KJFB30, KJFB43, KJFB56, KJFB30B, KJFH96, KJFH01, BNPA92, KJAH30, KJFH20, KJFH, KJAH33, KJFH10, KJ6B10, KJFH00 (*)Has to be registered accompanied by one of the following: DK650, DK650P, DK659, DK650M, DK650N, DK658 |
| Ischemic colitis | Categorical | Yes(1)/No(2) | When blood flow to part of the large intestine is reduced | LPR | DK550A In combination with KJFB-, KJFB20, KJFB96, KJA-, KJFB46, KJFB30, KJFB43, KJFB56, KJFB30B, KJFH96, KJFH01, KJH00, BNPA92, KJAH30, KJFH20, KJFH, KJAH33, KJFH10, KJ6B10, KJFH00 (*)Has to be registered accompanied by one of the following: DK650, DK650P, DK659, DK650M, DK650N, DK658 |
| Perforation after screening colonoscopy | Categorical | Yes(1)/No(2) | Perforation after colonoscopy | LPR | DT812G1 In combination with KJFB-, KJA-, KJFB20, KJFB96, KJH00, KJFB46, KJFB30, KJFB43, KJFB56, KJFB30B, KJFH96, KJFH01, KJH00, KJGB10, KJGB40, KJGB00, KJGB30, KJGB31, BNPA92, KJAH30, KJFH20, KJFH, KJAH33, KJFH10, KJ6B10, KJFH00 (*)Has to be registered accompanied by one of the following: DK650, DK650P, DK659, DK650M, DK650N, DK658 |
| Peritonitis | Categorical | Yes(1)/No(2) | Inflammation in the peritoneal cavity | LPR | DK659, DK650, DK650M, DK650N, DK658, DK650P In combination with KJFB-, KJFB96, KJFB20, KJFB96, KJH00, KJFB46, KJFB30, KJFB43, KJFB56, KJFB30B, KJFH96, KJFH01, KJA-, KJGB10, KJGB40, KJGB00, KJGB30, KJGB31, BNPA92, KJAH30, KJFH20, KJFH, KJAH33, KJFH10, KJ6B10, KJFH00 |

****Table S2**:** Logistic regression models for overall mortality: Comparison between VAC and PAC

| **Univariate** | **Odds ratio** | **Std. error** | **P-value** | **Lower CI** | **Upper CI** |
| --- | --- | --- | --- | --- | --- |
| **Procedure** |  |  |  |  |  |
| PAC | Ref | - | - | - | - |
| VAC | 0.89 | 0.058 | 0.068 | 0.78 | 1.1 |
| **Multivariate** |  |  |  |  |  |
| **Procedure** |  |  |  |  |  |
| PAC | Ref | - | - | - | - |
| VAC | 1.10 | 0.87 | 0.25 | 0.90 | 1.36 |
| **Age group** |  |  |  |  |  |
| >45 | Ref | - | - | - | - |
| 45-54 | 2.42 | 0.298 | <0.001 | 1.89 | 3.08 |
| 55-64 | 4.04 | 0.452 | <0.001 | 3.24 | 5.03 |
| 65-74 | 5.90 | 0.643 | <0.001 | 4.77 | 7.31 |
| >74 | 16.58 | 1.822 | <0.001 | 13.37 | 20.57 |
| **Sex** |  |  |  |  |  |
| Men | Ref | - | - | - | - |
| Women | 0.69 | 0.03 | <0.000 | 0.64 | 0.76 |
| **Income quantile** |  |  |  |  |  |
| 1 | Ref | - | - | - | - |
| 2 | 0.95 | 0.057 | 0.386 | 0.84 | 1.01 |
| 3 | 0.68 | 0.041 | <0.001 | 0.61 | 0.77 |
| 4 | 0.46 | 0.031 | <0.001 | 0.45 | 0.53 |
| **Highest completed education** |  |  |  |  |  |
| Elementary | Ref | - | - | - | - |
| Vocational/high school | 0.88 | 0.042 | 0.005 | 0.79 | 0.96 |
| Short-intermediate | 0.77 | 0.052 | <0.001 | 0.68 | 0.88 |
| Longer | 0.80 | 0.086 | 0.038 | 0.65 | 0.99 |
| **Charlson Comorbidity Index** |  |  |  |  |  |
| 0 | Ref | - | - | - | - |
| 1 | 2.35 | 0.163 | <0.001 | 2.05 | 2.69 |
| 2 | 2.73 | 0.14 | <0.001 | 2.45 | 3.01 |
| 3 | 6.46 | 0.39 | <0.001 | 5.74 | 7.27 |
| **Year of Surgery** |  |  |  |  |  |
| 2007-2014 | Ref | - | - | - | - |
| 2015-2021 | 0.27 | 0.012 | <0.001 | 0.25 | 0.30 |

Logistic regression models show ORs, Std. error, p-values, and 95% CIs for univariate and multivariate analyses. The dependent variable in both univariate and multivariate models is overall mortality. The multivariate model adjusts for procedure, age, sex, income, education, CCI, and year of surgery.

**Abbreviations:** PAC, primary abdominal closure; VAC, vacuum-assisted closure; OR, odds ratio; Std. error, standard error; CI, confidence interval; CCI, Charlson Comorbidity Index.

**
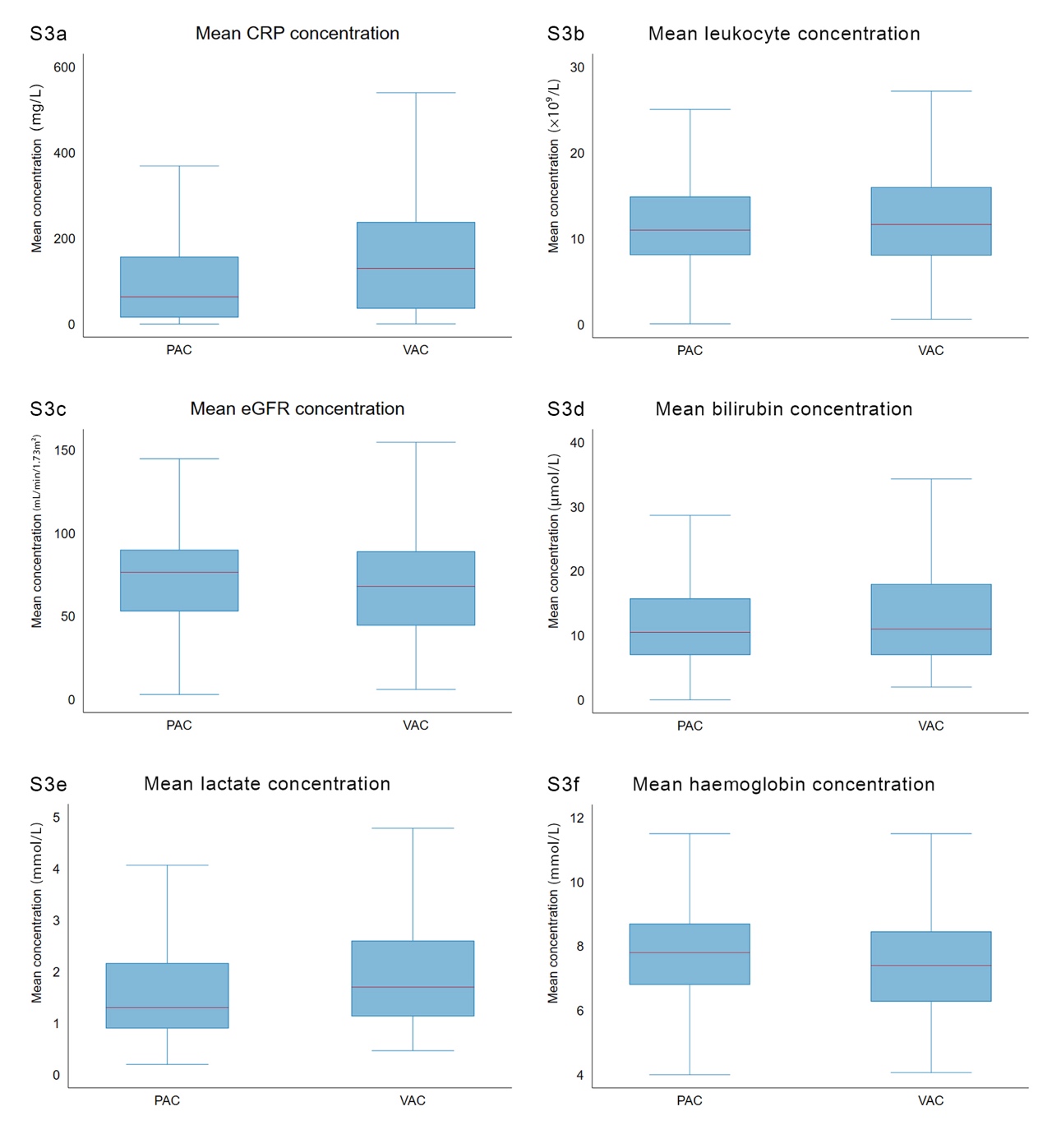
**

**Figure S3:** Boxplots depicting the distribution of mean concentrations of six biochemical markers in PAC and VAC groups. Subplots S3a–S3f show CRP (mg/L), leukocytes (×10⁹/L), eGFR (mL/min/1.73m²), bilirubin (µmol/L), lactate (mmol/L), and haemoglobin (mmol/L), respectively. Boxes represent the interquartile range (IQR), the horizontal line within each box indicates the median, and whiskers extend to the most extreme values within 1.5 times the IQR. Outliers beyond this range are excluded.


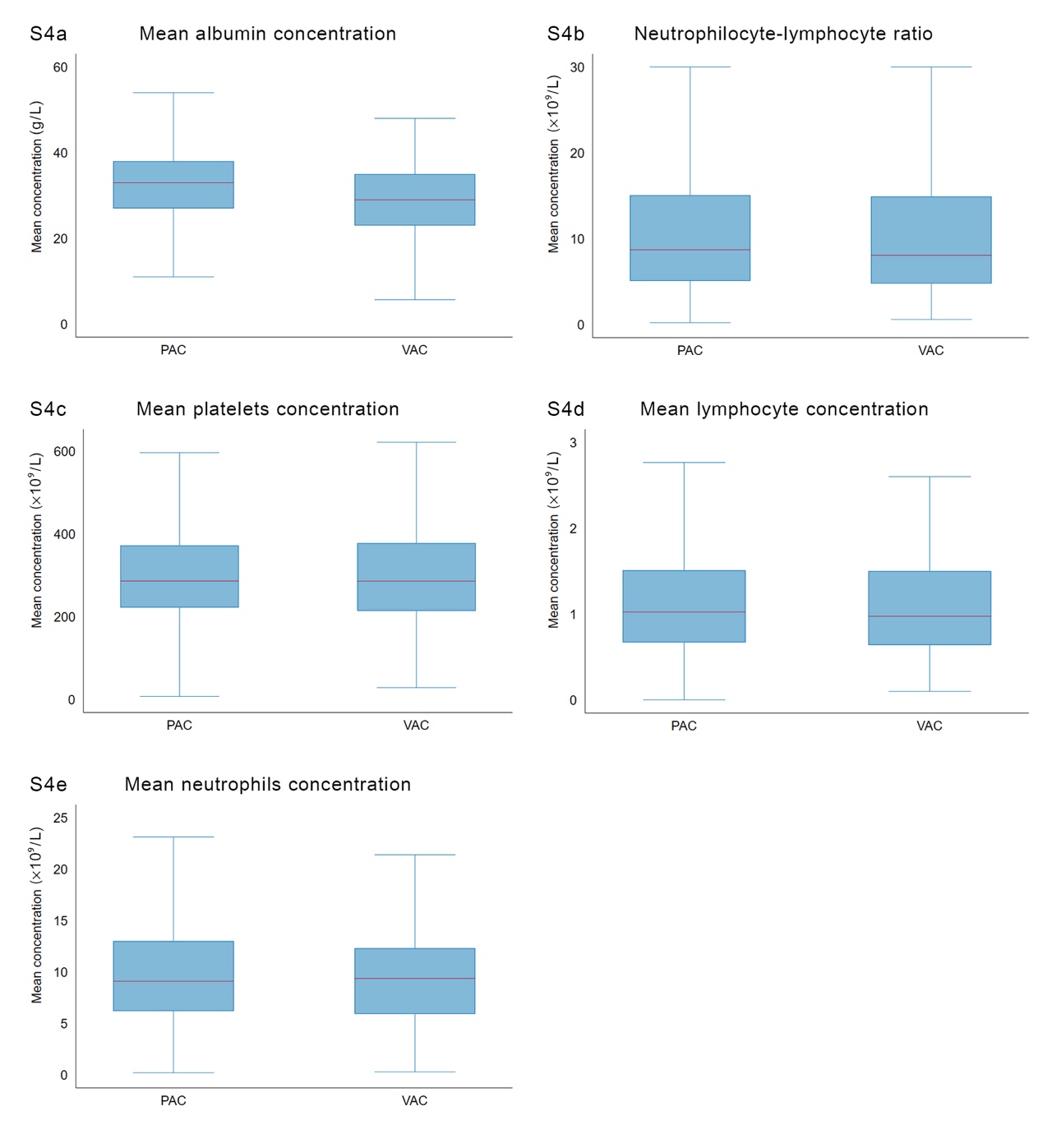


**Figure S4:** Boxplots depicting the distribution of mean concentrations of five immunologic and hematologic markers in PAC and VAC groups. Subplots S4a–S4e show albumin (g/L), neutrophil-to-lymphocyte ratio, platelets (×10⁹/L), lymphocytes (×10⁹/L), and neutrophils (×10⁹/L), respectively. Boxes represent the interquartile range (IQR), the horizontal line within each box indicates the median, and whiskers extend to the most extreme values within 1.5 times the IQR. Outliers beyond this range are excluded.
